# The Intricate Role of p53 in Adipocyte Differentiation and Function

**DOI:** 10.3390/cells9122621

**Published:** 2020-12-07

**Authors:** Yun Kyung Lee, Yu Seong Chung, Ji Hye Lee, Jin Mi Chun, Jun Hong Park

**Affiliations:** 1 Department of Internal Medicine, Seoul National University Bundang Hospital, Seongnam 13620, Korea; leeykyung@gmail.com; 2 Herbal Medicine Resources Research Center, Korea Institute of Oriental Medicine, Naju-si 58245, Korea; chung216@kiom.re.kr (Y.S.C.); 2jh@kiom.re.kr (J.H.L.); jmchun@kiom.re.kr (J.M.C.)

**Keywords:** p53, adipogenesis, white adipocytes, brown adipocytes, differentiation

## Abstract

For more than three decades, numerous studies have demonstrated the function of p53 in cell cycle, cellular senescence, autophagy, apoptosis, and metabolism. Among diverse functions, the essential role of p53 is to maintain cellular homeostatic response to stress by regulating proliferation and apoptosis. Recently, adipocytes have been studied with increasing intensity owing to the increased prevalence of metabolic diseases posing a serious public health concern and because metabolic dysfunction can directly induce tumorigenesis. The prevalence of metabolic diseases has steadily increased worldwide, and a growing interest in these diseases has led to the focus on the role of p53 in metabolism and adipocyte differentiation with or without metabolic stress. However, our collective understanding of the direct role of p53 in adipocyte differentiation and function remains insufficient. Therefore, this review focuses on the newly discovered roles of p53 in adipocyte differentiation and function.

## 1. Introduction

The multifunctional protein p53 (encoded by TP53 in humans and Trp53 in mice, hereafter termed p53) is a key regulator of cell proliferation and apoptotic response to exogenous or endogenous cellular stresses [1]. Under various cellular stresses, p53 protects normal cells against tumorigenesis in response to oncogene activation, dysregulation of the cell cycle, ribosomal stress, oxidative stress, DNA damage, and hypoxia [2]. p53 is involved in several intricate signaling pathways including those that regulate gene expression, protein stability, protein interaction, and post-transcriptional modifications [3]. In response to cellular stress, p53 contributes to various cellular physiological functions. Its concentration or phosphorylation status directly regulates essential physiological functions. It also regulates metabolic homeostasis, mitochondrial function, and antioxidant defense mechanisms [4,5,6,7]. Under mild or no stress, p53 controls cellular physiological activities by regulating the energy status of the cell and availability of nutrients, growth factors, and hormones [4,6,8,9]. It also regulates antioxidant gene functions [4,10,11]. Because of these multifunctional roles of p53, although p53 knockout mice are viable, a significant number of p53 knockout mice die during embryogenesis because of developmental problems including defects in embryo implantation, the neuronal system, and serious multiple congenital abnormalities [12,13]. However, the role of p53 in specific research areas such as aging is still unclear. For example, p53 levels were found to be increased in a pro- and anti-aging mouse model [14].

In the absence of cellular stresses, mouse double minute 2 (MDM2), an E3 ubiquitin ligase, regulates p53 levels via proteasomal degradation pathways [15]. When cells are exposed to stress, ataxia–telangiectasia-mutated kinase phosphorylates p53, which forms the p53-MDM2 complex [16]. When the p53 pathway is activated, the total level of p53 and its binding activity rapidly increases after phosphorylation. Activated p53 further activates several genes whose products induce cell cycle arrest, apoptosis, DNA repair, cellular senescence, and metabolic reprogramming [17,18]. p53 activity should be tightly regulated to allow normal cell growth and development because its activation prevents cell growth and proliferation. Interestingly, several studies have suggested that p53 regulates the balance between cellular development and differentiation. Schmid et al. demonstrated that p53 is highly expressed in midgestation mouse embryos and its level decreases after organogenesis [19]. However, the contribution of p53 in differentiation or development in a mouse model is still debatable [20].

Adipose tissue plays an important role in the regulation of systemic energy balance and glucose homeostasis. For example, adipose tissues regulate energy storage, lipid metabolism, glucose homeostasis, and energy expenditure [21]. The epidemic of metabolic disorders has presented a serious public health concern worldwide. Hence, adipocyte differentiation needs to be thoroughly studied. Three types of adipose tissues are found in mammals including humans: white adipose tissue (WAT), beige (or brite) adipose tissue, and brown adipose tissue (BAT) [22]. Although all adipose tissues are derived from mesenchymal progenitor cells, specific proteins or transcriptional factors regulate adipocyte lineage during adipogenesis in vivo and in vitro [23,24,25]. On embryonic day 14.5 and 15.5 post-coitus, BATs develop from early muscle progenitor cells that express myogenic transcription factors (Myf5) and BATs located around the central organs in mouse model [23]. BATs are rich in mitochondria and express uncoupling protein 1 (UCP1). They use substrates such as fatty acids and glucose to generate heat in response to various stimuli such as cold. Because of the function of non-shivering thermogenesis, BATs are considered a novel therapeutic target for treating metabolic diseases such as obesity.

On the other hand, the main function of WATs is energy storage in the triglyceride form. WATs are generally located subcutaneously throughout the body and constitute as much as 20–30% of the body weighty of normal humans. WAT progenitor cells are differently developed according to location; subcutaneous white adipose tissues develop before birth but visceral white adipose tissues develop after birth [26,27]. However, none of the WATs have the same characteristics. Specific WATs are incredibly dynamic and respond to stimuli including internal or external signaling and metabolic demands. Subcutaneous WATs are more prone to browning than visceral adipose tissues, and they can be activated upon exposure to cold temperatures or β-adrenergic stimulation because subcutaneous WATs are initiated from different lineage progenitor cells compared to visceral adipose tissues [28]. These browning adipocytes that appear in WATs are called “beige” adipocytes, which are induced by the browning stimulus leading to phenotypical similarity with the classical BAT such as abundance of respiratory mitochondria, multilocular small lipid droplets, and increased expression of Ucp1. WAT adipose tissues increase in cell number and size after weaning [29]. The mature white adipocyte is filled with a single lipid droplet containing a few mitochondria. Recent studies have demonstrated that p53 levels in WATs are increased in diet-induced or genetic obesity mouse models [30,31]. Hence, determining the role of p53 in the regulation of adipogenesis and cellular metabolism could reveal an important association between obesity and cancer.

In this review, we discuss the important role of p53 and highlight the key molecules that are regulated by p53 during adipogenesis. We also discuss the role of p53 in regulating metabolism in adipose tissues. Finally, we identify important outstanding questions and issues pertaining to the role of p53 in adipogenesis that remain to be addressed.

## 2. Role of P53 and White Adipocyte Differentiation and Lipid Metabolism

p53 is a regulator of cell survival and proliferation in response to multiple stress signals against abnormal cell growth and tumorigenesis. Apart from its role as a tumor suppressor, p53 has been involved in the regulation of differentiation of mesenchymal cells that can suppress white adipocyte differentiation [32,33,34]. White adipocytes store and accumulate lipids throughout their differentiation cycle. Adipocytes are derived from multipotent mesenchymal stem cells (MSCs), which are first committed to the adipogenic lineage and then transformed into preadipocytes. In secondary terminal differentiation, preadipocytes become mature adipocytes. The process of adipocyte differentiation involves three defined stages. The first step is the commitment of MSCs to the adipocyte lineage. The second step is mitotic clonal expansion (MCE) involving DNA replication and duplication of cells. During the early phase of differentiation, cell-cycle-arrested cells re-enter the cell cycle and undergo one or two rounds of the cell cycle, which is regarded as MCE. Given the association of cell cycle regulation with adipogenesis, the inhibitory effects of p53 in white adipocyte differentiation might occur via regulation of expression of several genes including p21 [35]. For example, MEFs lacking either p53 or p21 undergo spontaneous adipogenesis, and mice lacking p21 and p27 show adipose tissue hyperplasia [36,37]. The inhibitory effect of p53 on adipocyte differentiation relies on its transcriptional activity. Inhibition of adipocyte differentiation might result from forced expression of p53 during mitotic clonal expansion. Thus, the possible mechanism linking clonal expansion to adipogenesis is the capability of p53 to suppress adipogenesis and is dependent on its DNA binding ability, demonstrated by the failure to inhibit MEF adipogenesis by p53 with a mutation in the DNA-binding domain [38,39]. The third step is terminal differentiation, which involves transcriptional factors such as the CCAAT/enhancer-binding proteins (C/EBPs) family and peroxisome proliferator-activated receptor–γ (PPARγ), and a significant expression of lipogenic genes such as adiponectin and fatty acid-binding protein 4 (Fabp4, also called aP2) [40,41,42]. Huang et al. demonstrated that p53 partially suppressed preadipocyte differentiation and adipogenesis in the process of regulating adipogenic gene expression and Akt signaling [34]. Therefore, p53 protein is involved in white adipogenesis and plays a specific role in the regulation of adipogenesis. Although the role of p53 as a tumor suppressor has been clearly established, the transcriptional activity of p53 during adipogenic differentiation has not been adequately studied.

**Figure 1:**
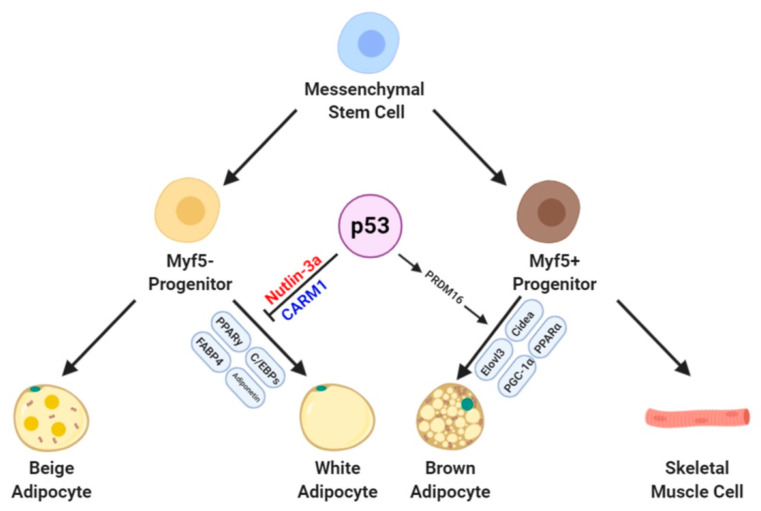
Proposed hypothetical model of the transcriptional role of p53 in white and brown adipogenesis.

Adipogenesis is controlled by several transcriptional factors including C/EBPs and PPARγ. C/EBPβ and C/EBPδ are expressed at an early stage of adipocyte differentiation and they induce the expression of C/EBPα and PPARγ [40,41,42]. In murine preadipocyte cell lines such as 3T3-L1, nutlin-3a-mediated p53 protein accumulation, and ectopic p53 overexpression cause a decrease in the expression of Pparγ and its coactivator PPARγ coactivator1 alpha (Ppargc1a or Pgc-1α) [32,34,43]. Knockdown of p53 in differentiated 3T3-L1 cells increases Pgc-1α gene expression. Furthermore, a few studies have reported enhanced accumulation of lipid droplets with an increase in Pparγ and aP2 protein levels [32,43]. The mesenchymal stem cell line C3H10T1/2 can differentiate into white adipocytes upon induction of adipogenesis. Compared to 3T3-L1 cells, the C3H10T1/2 cell line has been used to investigate early events of adipogenesis [44,45]. Knockdown of p53 in C3H10T1/2 cells was enhanced by the upregulation of adipogenic genes such as Pparγ, C/ebpα, aP2, and adiponectin [32]. Furthermore, knockdown of p53 by specific shRNA enhanced the adipogenic capacity in both mouse and human cell lines, in which the levels of adipogenic gene markers such as PPARγ, aP2, and Adipoq increased [32,33]. In addition to cell lines, mouse embryonic fibroblasts (MEFs) isolated from p53 knockout mice were differentiated into adipocytes. The MEFs obtained from p53 knockout mice accumulated more lipid droplets and showed increased expression of adipogenic markers such as Pparγ and C/ebpα compared with wild-type MEFs [32,33]. In addition, nutlin is known to induce stabilization and activation of p53 [46]. Nutlin-3a-mediated p53 increment led the downregulation of Pparγ expression in wild-type MEFs [33]. In line with the inhibitory role of p53 in adipocyte differentiation, the expression of p53 was downregulated in primary differentiated cells derived from the stromal vascular fraction of inguinal WAT (iWAT) obtained from p53 knockout mice and human adipocyte-derived stem cells with knockdown of p53 [32,47]. Moreover, coactivator-associated arginine methyltransferase 1 (Carm1) has been shown to enhance adipogenesis by activating PPARγ in 3T3-L1 cells [43]. Thus, CARM1 has also been suggested as a possible modulator of the anti-adipogenic effects of p53. Furthermore, p53 activation following nutlin-3a treatment in 3T3-L1 cells reduced adipocyte development and downregulated *Carm1* and reduced its protein expression (Figure 1). Importantly, overexpression of CARM1 prevented the inhibition of adipocyte differentiation after nutlin-3a treatment [48,49]. Another p53 target protein is twist family BHLH transcription factor 2 (Twist2). Twist2 is an essential protein that regulates adipocyte differentiation in marrow-resident MSCs. Loss of p53 induces the exhaustion of reactive oxygen species (ROS) in mitochondria and downregulates the expression of Twist2. These phenomena block the adipogenic differentiation and lead to osteogenic differentiation in MSCs. These results indicate that p53 is important not only for adipogenesis but also to maintain MSC integrity [50]. Lastly, the amount of Mdm2 is also important. Mdm2 is a major negative regulator of p53 and is highly expressed in 3T3-L1 preadipocytes [51]. However, Mdm2-p53 double knockout mouse embryonic fibroblast is not able to differentiate into mature adipocyte, and Mdm2 is known to regulate adipocyte differentiation in a p53-independent manner [52,53,54]. Therefore, as a negative regulator of p53 in 3T3-L1 adipogenesis, the role of Mdm2 needs to be carefully considered.

The canonical pathway of adipocyte lipolysis is catalyzed by three main lipases [53,54]. The first lipase is called patatin-like phospholipase domain containing-2 (PNPLA2)/adipocyte triglyceride lipase (ATGL), which is the rate-limiting enzyme for triacylglycerol (TAG) hydrolysis. The cholesterol esterase called “hormone-sensitive lipase” can utilize multiple substrates such as TAG, DAG, and retinyl. The third lipase is monoacylglycerol lipase (MGL). These three lipases function together to degrade TAGs sequentially, resulting in the release of three types of fatty acids (FAs) and glycerol. Additionally, adipocyte lipolysis involves multifunctional enzymes that catalyze reactions in both directions, resulting in the re-esterification and recycling of FAs [55,56]. The role of p53 in white adipocytes extends beyond the regulation of adipogenesis. p53-associated lipolysis could control the regulation of genes involved in lipid droplet accumulation [57]. In human adipocytes, overexpression of p53 reduces lipid droplet accumulation. Furthermore, inhibition of lipolysis by acipimox and induction of lipolysis by isoproterenol showed that p53 expression and lipolysis were closely regulated in mouse WATs [58]. Furthermore, Andreas et al. showed that DNA damaged induced transcript 4 (Ddit4), which is upregulated by fasting in white adipose tissue, is a p53 target gene. Ddit4 is induced by p53 activation and its ectopic expression augments lipolysis in differentiated C3H10T1/2 adipocytes [59]. Most recently, Wang et al. demonstrated that ATGL promoter has a conserved p53 binding site that promotes p53-induced ATGL promoter activity and transcription. Therefore, p53 induces lipolysis by directly activating ATGL transcription [60].

Recent studies have suggested that activation of p53 under abnormal metabolic conditions in adipose tissues aggravates obesity. Kung et al. demonstrated that mice harboring the proline-to-arginine 72 (P72R) variant of p53 who were administered a high-fat diet developed severe obesity and metabolic dysfunction than mice expressing a proline 72 variant [61]. In line with these results, p53 inhibition is known to prevent weight gain against a high-fat diet in mouse model [62]. Altogether, these results indicate that p53 regulates lipid metabolism in adipocytes. Taken together, these studies have suggested that p53 plays an important role in white adipocyte differentiation and function.

## 3. Role of P53 in Brown Adipocyte Differentiation and Thermogenesis

BATs are located around the central organs and are essential for whole-body energy homeostasis, particularly energy expenditure [63,64]. Hence, BATs have been suggested to be a novel therapeutic target for the treatment of diabetes and obesity [22]. Because BATs are important for cold-induced, nonshivering thermogenesis in newborn mouse pups the generation of BATs begins in the mid/late postnatal stage and undergoes rapid development [23,65]. Although BATs and WATs have different developmental origins and physiological functions, they share many common adipogenic developments including a conserved PPARγ and C/EBPα-driven transcriptional regulation of adipogenesis. Several recent studies have revealed that p53 plays a specific role in brown adipogenesis both in vitro and in vivo. We discuss these roles of p53 in brown adipogenesis in the subsequent sections.

A recent study performed by Molchadsky et al. demonstrated that p53 is a positive regulatory factor that maintains proper brown adipocyte maturation in vitro and prevents the development of obesity because of a high-fat diet in the mouse model [32]. Loss of p53 in the PR-domain contains 16 (PRDM16)- overexpressing myogenic C2 cells reduced the expression of general adipogenic genes and functional brown marker genes such as aP2, cell death-inducing DFFA-like effector a (Cidea), and ELOVL fatty acid elongase 3 (Elovl3). In addition, oil red O staining results support the assumption that p53 is required for the normal development of brown adipocytes [32]. Consistent with these results, the depletion of p53 downregulates PRDM16 expression. PRDM16 is a key transcriptional regulator in determining the brown adipocyte lineage and their subsequent development [66,67]. PRDM16 consists of a PR/SET domain at the N-terminal, two zine-finger domains containing seven or three C2H2 zinc finger domains, a proximal regulatory region, a repression domain, and an acidic activation domain in the C-terminal [68]. Because PRDM16 can bind directly to a specific DNA sequence via two sets of zinc finger domains or form a protein-protein complex with other adipogenic regulators, it regulates transcriptional activities in BAT development. PRDM16 knockout has been shown to induce a significant reduction in BAT development in a mouse model [69]. Moreover, PRDM16 regulates not only differentiation but also functional genetic expression in brown adipocytes. The expression of PRDM16 controls the transcription of essential brown adipocyte functional genes such as Elovl3, Cidea, Pgc-1α, and PPARα [70,71,72]. Cidea and Pgc-1α are important transcriptional regulators of brown adipocyte thermogenesis, which are involved in the regulation of *Ucp1* transcriptional activity [73,74]. Overexpression of PRDM16 could upregulate the expression of genes whose products are involved in the mitochondrial oxidative pathway in mature brown adipocytes [69]. In vivo studies have shown that depletion of p53 induces abnormal BAT differentiation and is unprotected against diet-induced obesity [32]. This phenomenon may be caused by the loss of p53 could not suppress the expression of PPARγ in WATs and induction of energy expenditure in BATs. Consistent with these findings, Gan et al. demonstrated that Forkhead box C2 (Foxc2) suppressed differentiation and enhanced proliferation in preadipocytes [75]. Foxc2 is a forkhead/winged-helix transcription factor family protein. It plays an important role in cell growth, proliferation, differentiation, apoptosis, longevity, and energy metabolism [76,77]. Gan et al. showed that feeding a high-fat diet upregulated the expression of *Foxc2* in mouse preadipocytes, which increased the expression of cyclin E and inhibited that of p53 and p27 in preadipocytes (Figure 1).

On the other hand, Hallenborg et al. showed that depletion of p53 in mice protected them against high-fat diet-induced obesity [47]. Loss of p53 increased the transcriptional activity of Pgc-1α and Pgc-1β and upregulated the expression of Ucp1 in MEF cells derived from p53 mutant mice. Importantly, they also showed that BATs from the p53 mutant mice were phenotypically different than the regular BAT, and that p53 mutant-expressing brown adipocytes more closely resemble beige adipocytes. Consistent with these results, whole p53 KO mice fed a high-fat diet show less body weight gain than WT littermates; however, conditional genetic mutation of p53 in BAT did not affect body weight gain or thermogenic activity [78]. Although this interesting study showed controversial phenotypes in mouse models fed a high-fat diet compared to other studies, these results suggested that p53 plays a critical role in brown adipocyte differentiation.

## 4. Role of P53 in Beige Adipocytes and Browning

p53 has been known to be involved in the aging process of WATs [79]. The total level of p53 has been found to be increased in the WATs of aged mice, and increased p53 level induces the expression of p21 in WATs, which further increases the release of proinflammatory cytokines and causes insulin resistance [79]. Furthermore, a recent study demonstrated that p53 regulates the browning of iWAT [80]. Elevated p53 suppressed the mitochondrial increase during browning in iWATs. In addition, they showed that transient depletion of p53 in the adipose tissue-specific p53 conditional knockout mouse model or using a pharmacological inhibitor (Pifithrin-α) restored cold-induced iWAT browning and whole-body energy metabolism in aged mice [80]. It is also possible that p53 regulates *PRDM16* expression via interaction with PRDM16 or binding to the PRDM16 promoter [32,47]. However, the definitive role of p53 in beige adipogenesis and browning will require further study.

## 5. Conclusions

Over the past four decades, the roles of p53 have been extensively studied because the mutation or inhibition of p53 initiates tumorigenesis [1,14,81,82,83]. p53 has been found to be regulated by various stress signaling pathways and controls the cellular homeostasis response to stress [1,2]. p53 is the control tower of both intra- and extracellular stresses that activate various p53 pathways, which affects multidirectional physiological functions in various tissues. Recently, interesting studies using mouse models have demonstrated that p53 not only responds to cellular stress but is also involved in tissue or organ differentiation [81]. Recent advancements in determining the role of p53 in developmental biology have suggested its fundamental role in controlling cellular differentiation and why we need to improve our understanding of the different roles of p53. The newly identified biological roles of p53 in the regulation of adipogenesis and metabolic function have enabled researchers to study important and previously unexplored aspects of p53 function. For example, how do cancer cells affect metabolism or body fat mass in cancer patients? Depending on the cancer type, 30–80% of cancer patients develop cancer-associated cachexia (CAC) in the last stage of malignancy. Cancer cachexia leads to atrophy of adipose tissue and/or metabolic dysfunction, which accelerates the progression of cancer. Cancer cells secrete many inflammation mediators including nuclear factor kappa-light-chain-enhancer of activated B cells, tumor necrosis factor-a, interleukin-1, and interleukin-6 that have been shown to increase the metabolic expenditure through the activation of thermogenesis, inhibit differentiation of muscle and adipose tissues, and cause loss of appetite [84,85,86]. These inflammatory mediators can suppress the transcriptional activity of p53, and abnormal regulation of p53 could affect the initiation or progression of CAC [87]. A better understanding of p53 in regulating adipose tissue development and function will lead to the identification of novel therapeutic targets for CAC.

Heretofore, p53 role in adipogenesis, especially white adipocytes, is a negative regulator of adipogenesis. However, the role of p53 in negative regulation of adipogenesis under exact molecular upstream and downstream networks remains unknown. Generally, p53 is considered a stress response protein. WATs have been considered to be simple energy storage tissues containing lipid droplet organelles but they also have endocrine functions and secrete several factors such as adipokines [21]. The association between adipose tissues and p53 has become increasingly evident in the last few years. For example, excessive calorie intake could enhance the generation of reactive ROS in the adipose tissues and cause DNA damage, which could activate p53 [58,79]. Exogenous stress such as cardiac pressure can activate p53 in adipose tissue. Activation of the sympathetic nervous system upregulates adipose tissue p53 through increasing lipolysis, and the inhibition of p53 attenuates adipose tissue inflammation [58]. However, the upstream signals that activate p53 to induce stress signals remain unclear. The question remains as to whether p53 activation is stress-induced or depends on the tissue environment? Also, the environment in which p53 regulates lipid metabolism needs to be determined. Although several in vivo and in vitro studies have demonstrated the role of p53 in white adipocyte differentiation and function, the direct role of p53 in WAT formation needs to be determined. Since mutation of p53 results in the reduction of cell proliferation or growth, further investigation is needed to distinguish the direct role of p53 in the cellular proliferation and differentiation of adipocytes.

One of the major unsolved questions is the direct role of p53 in brown adipogenesis. Previously performed studies have reported contrasting results regarding the role of p53 in brown adipogenesis. Inhibition of p53 suppressed brown adipogenesis in Prdm16-overexpressed myogenic C2 cells in vitro [32]. On the other hand, p53 depletion enhanced the expression of brown adipogenic genes in MEFs and primary adipocytes derived from p53 knockout mice [47]. Similarly, the same mouse model showed different phenotypes in the high-fat diet experiment. These results have caused a lack of clarity in understanding the direct role of p53. Why did the depletion of p53 lead to the development of different phenotypes? The first reason may be the difference in cellular characteristics. Although these cells were derived from the same progenitor cells, different cellular characteristics may affect the response of p53 or cellular development. A pioneering study has suggested that brown adipocytes arise from the same progenitor cells as the skeletal muscle that expresses the transcription factor Myf5 [69]. Furthermore, this study demonstrated that brown adipocytes share many features with skeletal myocytes, and that loss of PRDM16 induces muscle differentiation in brown adipocyte progenitors. Supporting this hypothesis, loss of Ewing’s sarcoma breakpoint region 1 blocks maturation of brown adipocytes and induces myogenic differentiation in brown preadipocytes [23]. Physiologically, skeletal myocytes are rich in mitochondria and are specialized in lipid catabolism rather than lipid storage similar to brown adipocytes. Although skeletal myocytes share many features with brown adipocytes, they exhibit different characteristics. For example, they can express Pgc-1α; however, myocytes do not express Ucp1. These differences induce a different response to the expression of adipogenic genes. The second reason is the unique characteristic of p53, which has specific roles dependent on tissue or development stage. Omar Al-Massadi et al. showed that pharmacological or genetic mutation of p53 in BAT affects thermogenic programs and body weight in adult male mice, but not in embryos [78]. Additionally, they showed that pharmacological or genetic stimulation of p53 in the BAT of obese mice protects them from obesity and induces thermogenic activity. The third reason may be the original role of p53. p53 regulates endogenous or exogenous cellular stress. Hallenborg et al. suggested that housing temperatures, housing styles (group or individual), or differences in the gut microbiota could affect these contradictory results [47]. However, it is notable that normal housing temperatures could not suppress Ucp1 expression in p53-deficient mice [47]. These differences may be due to differences in behavior and mouse housing styles. Locomotion activities (horizontal, total distance traveled, number of movements, stereotypy counts) were significantly increased in neuronal-specific conditional p53 KO mice compared to wild-type mice [88]. In addition, individually housed mice had high nest scores, low body weight, and increased sucrose and food consumption [89]. These multiple factors may affect the contradictory phenotypes of p53 mice. However, further studies are needed to clarify these contradictory results.

The other mechanism by which p53 regulates cellular differentiation involves its chromatin-binding activity or interaction with specific protein partners. As described above, p53 affects adipogenesis dependent cell types in vitro. These results suggest that promoter remodeling following p53 binding to chromatin occurs at different sites in different adipogenic stages or binding with different protein partners in each cell. BATs and WATs share many essential adipogenic regulatory transcriptional factors including PPARγ and CEBPα. If p53 regulates promoter remodeling in the same way, there is no different response to adipogenic development regardless of the cell type. p53 interacts with chromatin response to diverse signaling and regulates transcription of specific target genes with or without co-binding factors [90,91,92]. Supporting this possibility, Chip analysis results showed that p53 binds to PRDM16 promoter regions in primary brown adipocytes [32]. In addition, global protein-protein interaction analysis by proteomics screening showed that the p53-Prdm16 or p53-Pgc-1a protein complex regulates adipocyte differentiation or function [47,93].

The role of p53 in tumorigenesis has been extensively studied because the activation of p53 kills cancer cells [94]. Many researchers have screened to find p53 modulator from herbal medicines to single molecules. However, due to multi-functional roles of p53 in diverse tissues, keeping a homeostatic balance of p53 is most important. Recently, studies have suggested that herbal medicines or single compounds from natural products might modulate p53 [95]. Nontoxic herbal medicines from plants, fruits, insects, and animals can provide novel p53 modulators. In this short review, we highlighted the roles of p53 in WAT and BAT adipogenesis and function. It is now important to carefully study the role of p53 in adipocytes. In the case of metabolic dysfunction, essential tissues are exposed to chronic cellular stress, and abnormal adipogenesis or metabolic dysfunction resulting from activation of p53 can cause more severe chronic stress. Considering the unique function of BATs, research on the role of p53 in these tissues could provide a novel therapeutic approach for treating metabolic disease. Although there are still many unanswered questions and contrasting results, this review highlights and explains the important role of p53 in adipocytes.
